# Hypoxia-inducible factor-2α stabilizes the von Hippel-Lindau (VHL) disease suppressor, Myb-related protein 2

**DOI:** 10.1371/journal.pone.0175593

**Published:** 2017-04-10

**Authors:** Fumihiko Okumura, Akiko Joo-Okumura, Kunio Nakatsukasa, Takumi Kamura

**Affiliations:** Division of Biological Science, Graduate School of Science, Nagoya University, Aichi, Japan; University of Dundee, UNITED KINGDOM

## Abstract

Ubiquitin ligase von Hippel–Lindau tumor suppressor (pVHL) negatively regulates protein levels of hypoxia-inducible factor-α (HIF-α). Loss of pVHL causes HIF-α accumulation, which contributes to the pathogenesis of von Hippel-Lindau (VHL) disease. In contrast, v-Myb avian myeloblastosis viral oncogene homolog–like 2 (MYBL2; B-Myb), a transcription factor, prevents VHL pathogenesis by regulating gene expression of HIF-independent pathways. Both HIF-α and B-Myb are targets of pVHL-mediated polyubiquitination and proteasomal degradation. Here, we show that knockdown of HIF-2α induces downregulation of B-Myb in 786-O cells, which are deficient in pVHL, and this downregulation is prevented by proteasome inhibition. In the presence of pVHL and under hypoxia-like conditions, B-Myb and HIF-2α are both upregulated, and the upregulation of B-Myb requires expression of HIF-2α. We also show that HIF-2α and B-Myb interact in the nucleus, and this interaction is mediated by the central region of HIF-2α and the C-terminal region of B-Myb. These data indicate that oncogenic HIF-2α stabilizes B-Myb to suppress VHL pathogenesis.

## Introduction

Ubiquitin-mediated proteolysis plays an important role in the elimination of specific proteins [1]. It involves two key steps: covalent attachment of multiple ubiquitin molecules to a target protein, and degradation of the polyubiquitinated protein by the 26S proteasome complex. The protein product of the von Hippel-Lindau (VHL) tumor suppressor gene, pVHL, forms a complex with elongin B and C, cullin2 (Cul2) and the RING finger protein, Rbx1 [2,3]. The pVHL complex polyubiquitinates the hypoxia-inducible factor-α (HIF-α) family of transcription factors (HIF-1-3α) for proteasomal degradation [4]. Hydroxylation of proline residues in LXXLAP sequence motifs of HIF-α proteins, which is mediated by prolyl hydroxylases (PHD1-3) under normoxic conditions, promotes polyubiquitination by pVHL [5,6] [7–9]. At low oxygen levels (i.e., hypoxia), HIF-α is not hydroxylated by PHDs, and is therefore not ubiquitinated by pVHL. Non-hydroxylated HIF-α dimerizes with HIF-1β, also known as aryl hydrocarbon receptor nuclear translocator (ARNT), and translocates to the nucleus to transcribe downstream target genes such as vascular endothelial growth factor A (VEGFA), solute carrier family 2 member 1 (SLC2A1, which encodes GLUT1), and platelet-derived growth factor (PDGF) [10]. Loss of functional pVHL or mutation of hydroxylatable prolyl residues in HIF-α prevents O_2_-dependent degradation of HIF-α, which results in constitutive expression of downstream HIF-dependent genes and VHL disease. VHL disease is characterized by a variety of lesions, including hemangioblastomas, clear cell renal cell carcinomas, pheochromocytomas, pancreatic islet cell tumors, endolymphatic sac tumors, and papillary cystadenomas of the broad ligament (females), and epididymis (males) [10].

B-Myb is targeted by pVHL ubiquitin ligase complex for proteasomal degradation [11]. Phosphorylation of tyrosine 15 on B-Myb by VEGF and/or PDGF prevents its ubiquitination and consequent degradation by pVHL. Since VEGF and PDGF gene expression is promoted by HIF, HIF pathway activation indirectly stabilizes B-Myb. B-Myb suppresses tumor growth induced by pVHL deficiency; larger tumors are formed *in vivo* by B-Myb-knockdown 786-O cells, which are deficient in pVHL, than by control cells. 786-O cells, which were established from a primary clear cell renal cell carcinoma, express HIF-2α at higher levels than HIF-1α [12]. HIF functions independently of B-Myb, as HIF-α-dependent genes are not affected by B-Myb knockdown [11]. We preliminarily found that knockdown of HIF-2α in 786-O cells reduces B-Myb protein levels; however, the mechanisms underlying this effect were not characterized. One possible mechanism is that HIF-2α interacts with B-Myb to prevent its degradation, but this has not yet been confirmed. Therefore, in the present study, we have investigated the interaction between HIF-2α and B-Myb. The data suggest that oncogenic HIF-2α stabilizes the VHL disease suppressor, B-Myb, by interacting with B-Myb.

## Results

### Knockdown of HIF-2α in 786-O cells downregulates B-Myb protein levels

**Fig 1:**
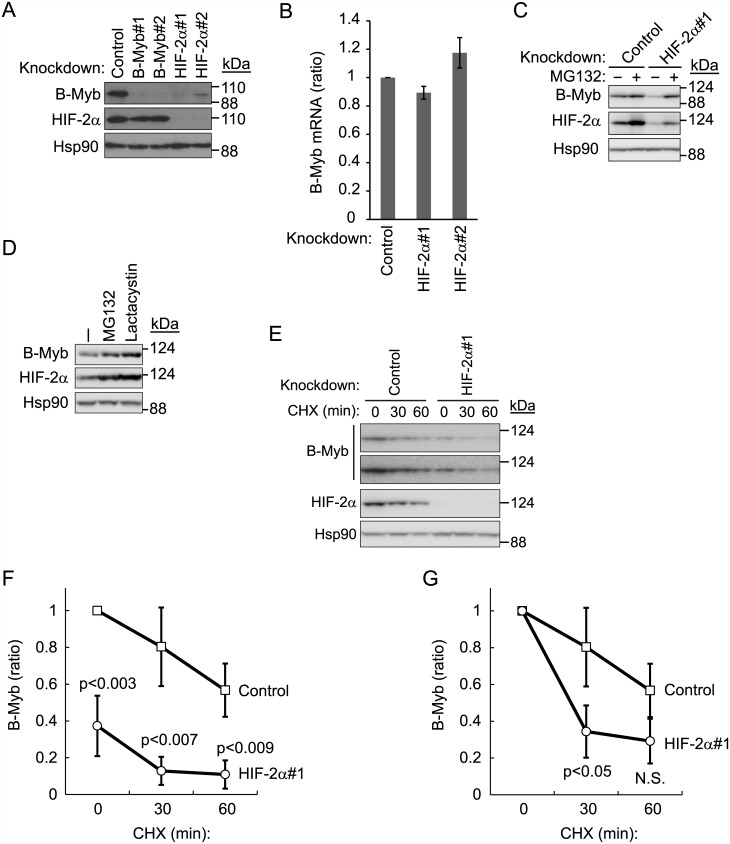
Knockdown of HIF-2α downregulates B-Myb in pVHL-deficient 786-O cells. (A) Knockdown of HIF-2α downregulates B-Myb protein levels in pVHL-deficient 786-O cells. Cell lysates of control-, B-Myb-, or HIF-2α-knockdown 786-O cell lines were immunoblotted with anti-B-Myb or anti-HIF-2α antibodies to determine protein levels of B-Myb and HIF-2α, respectively. Hsp90 was used as loading control. Representative data from three independent experiments are shown. (B) Knockdown of HIF-2α does not affect B-Myb mRNA levels. Total RNA was isolated from control- or HIF-2α-knockdown 786-O cells and analyzed by quantitative RT-PCR analysis. Data represent the mean ± SD of three independent experiments. (C) B-Myb downregulation by HIF-2α knockdown in pVHL-deficient cells is proteasome-dependent. Control or HIF-2α-knockdown 786-O cells were cultured in the presence or absence of MG132 (10 μM for 6 h) and subjected to immunoblotting with anti-B-Myb or anti-HIF-2α antibodies. Hsp90 was used as loading control. Representative data from three independent experiments are shown. (D) Stabilization of HIF-2α and B-Myb by MG132 and lactacystin. 786-O cells were cultured in the presence or absence of MG132 (10 μM for 6 h) or lactacystin (5 μM for 6 h) and immunoblotted with anti-B-Myb or anti-HIF-2α antibodies. Hsp90 was used as loading control. Representative data from three independent experiments are shown. (E) Stability of B-Myb with or without HIF-2α. Control 786-O cells or HIF-2α knockdown cells were exposed to cycloheximide (CHX, 50 μg/ml) for 30 or 60 min. The lysates were subjected to western blot with antibodies against B-Myb (short and long exposure are shown), HIF-2α, or Hsp90. Hsp90 was used as loading control. Representative data from three independent experiments are shown. (F) The intensities of the B-Myb bands in (D) were normalized to those of the corresponding Hsp90 bands and plotted as ratio of the normalized value against control cells at 0 min. Data are presented as the mean ± SD of three independent experiments. (G) The intensities of B-Myb bands in (D) at 0 min were set as 1.

In a previous study wherein the tumor-suppressive effect of B-Myb in VHL disease was investigated, we preliminarily found that knockdown of HIF-2α decreased protein levels of B-Myb in pVHL-deficient 786-O cells [11]. To validate this result, we independently knocked down HIF-2α with two distinct short hairpin RNAs (shRNA), which decreased B-Myb protein levels (Fig 1A). Knocking down B-Myb did not have a reciprocal effect on HIF-2α protein levels (Fig 1A). To determine whether HIF-2α directly regulates B-Myb transcription, we measured the mRNA expression of B-Myb after knocking down HIF-2α, and found no significant difference (Fig 1B). Thus, the B-Myb gene is not a direct target of HIF-2α. Although it is well known that pVHL targets HIF-2α for proteasomal degradation [13,14], HIF-2α protein levels increased even in pVHL-deficient 786-O cells after treatment with the proteasome inhibitors MG132 and lactacystin, suggesting that HIF-2α might be a substrate of other ubiquitin ligases (Fig 1C and 1D). B-Myb protein levels also increased in 786-O cells after MG132 or lactacystin treatment, suggesting that B-Myb is also regulated by ubiquitin-mediated proteolysis in the absence of pVHL, as suggested previously [11]. Importantly, proteasome inhibition with MG132 treatment nearly completely prevented the downregulation of B-Myb induced by HIF-2α knockdown (Fig 1C). MG132 treatment also partially rescued HIF-2α protein levels in cells treated with HIF-2α shRNA, although the rescue of HIF-2α protein levels was more modest compared to that of B-Myb protein levels (Fig 1C). Since the knockdown of HIF-2α was not complete, the degradation of residual HIF-2α might be blocked by MG132 treatment. Next, we compared the stability of B-Myb in the presence and absence of HIF-2α, using cycloheximide, which blocks protein translation (Fig 1E–1G). We previously reported [11] that B-Myb and HIF-2α are degraded in the absence of pVHL. Given that the B-Myb expression level in HIF-2α knockdown cells at the beginning of the chase experiment was lower than that in control knockdown cells, a comparison of the B-Myb half-life would have been inappropriate (Fig 1E and 1G). Nevertheless, on normalizing the B-Myb expression level to that at t0, the degradation rate was found to be higher in HIF-2α knockdown cells than in control cells (Fig 1G). We next examined the polyubiquitination level of endogenous B-Myb in control or HIF-2α knockdown cells, because knockdown of HIF-2α destabilizes B-Myb, and the polyubiquitination level of B-Myb was expected to increase. We performed immunoprecipitation using an anti-polyubiquitin antibody (FK2), followed by immunoblotting using an anti-B-Myb antibody to detect endogenous polyubiquitination of B-Myb. However, B-Myb polyubiquitination levels were below the detection level even in the presence of MG132 (data not shown). Reciprocal immunoprecipitation (IP: B-Myb, IB: ubiquitin) under denatured conditions to exclude polyubiquitination of proteins interacting with B-Myb was unsuccessful because the anti-B-Myb antibody was sensitive to denaturation. Although it is important to identify the ubiquitin ligases other than pVHL that mediate polyubiquitination of B-Myb and/or HIF-2α, we decided to focus on the stabilization of B-Myb by HIF-2α in this study, as this is an interesting, novel function of HIF-2α. Altogether, these data indicate that B-Myb is degraded by the proteasome in the absence of HIF-2α and pVHL, and suggest that HIF-2α stabilizes B-Myb.

### HIF-2α-dependent stabilization of B-Myb under hypoxic conditions

**Fig 2:**
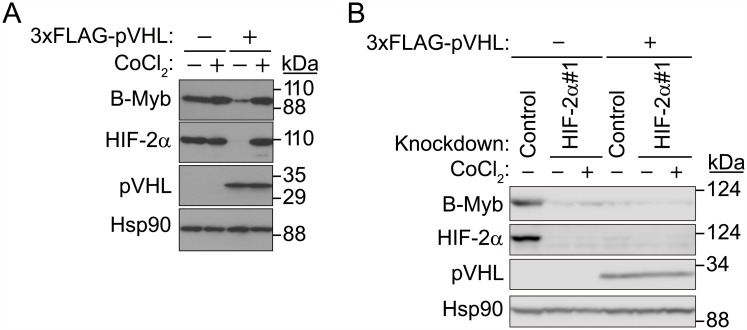
HIF-2α stabilizes B-Myb. (A) Control- or 3×FLAG-pVHL-expressing 786-O cell lines were cultured in the presence or absence of CoCl_2_ (400 μM) for 7 h, and cell lysates were immunoblotted with anti-B-Myb, anti-HIF-2α, or anti-pVHL antibodies. Forced expression of 3×FLAG-pVHL downregulates HIF-2α and B-Myb protein levels under normoxic conditions, and CoCl_2_ treatment rescues HIF-2α and B-Myb protein levels. Hsp90 was used as loading control. Representative data from three independent experiments are shown. (B) Knockdown of HIF-2α suppresses the accumulation of B-Myb under hypoxic-like conditions. Control or pVHL-expressing 786-O cell lines were transfected with non-specific or HIF-2α-targeting siRNA and cultured for 2 days, and cell lysates were immunoblotted with anti-B-Myb, pVHL, or anti-HIF-2α antibodies. Cells were incubated in the presence or absence of CoCl_2_ (400 μM) for 7 h before they were harvested. Hsp90 was used as a loading control. Representative data from three independent experiments are shown.

Under normoxic conditions, pVHL targets HIF-α for proteasomal degradation; however, under hypoxia, HIF-α escapes ubiquitination and degradation [4–9,11,13,14]. Since 786-O cells are pVHL-deficient, HIF-2α levels did not increase upon CoCl_2_ treatment. Since our initial results suggested that HIF-2α might stabilize B-Myb, we investigated whether B-Myb is also stabilized under hypoxic-like conditions in the presence of pVHL in 786-O cells when HIF-2α is stabilized (Fig 2A). pVHL-deficient 786-O cells, transfected either with control or 3×FLAG-tagged pVHL expression vectors, were treated with the hypoxia mimetic agent CoCl_2_ (400 μM) for 7 h under confluent conditions [11]. As expected, in 786-O cells transfected with control expression plasmids, B-Myb and HIF-2α were expressed at high levels (Fig 2A), and CoCl_2_ treatment did not affect the expression of either B-Myb or HIF-2α (Fig 2A). Under normoxic conditions, forced expression of pVHL downregulated both B-Myb and HIF-2α protein levels [4–9,11,13,14] (Fig 2A), and this effect was prevented with CoCl_2_ treatment (Fig 2A), suggesting that B-Myb and HIF-2α are similarly regulated under hypoxic-like conditions. In contrast, when HIF-2α was knocked down in pVHL-deficient and pVHL-expressing 786-O cells, B-Myb protein levels were significantly reduced under both normoxic and hypoxic-like conditions (Fig 2B). These data indicate that HIF-2α stabilizes B-Myb under hypoxic-like conditions only in the presence of pVHL.

### HIF-2α interacts with B-Myb

**Fig 3:**
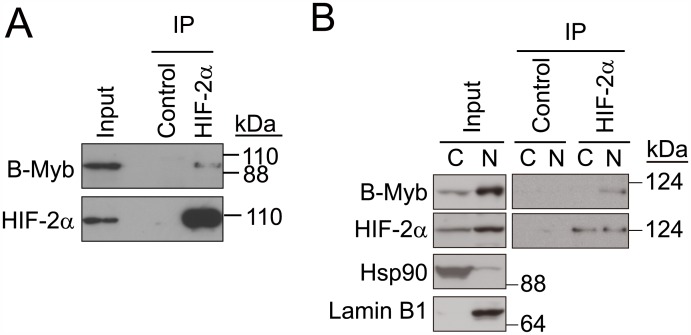
Endogenous nuclear HIF-2α and B-Myb interact. (A) Endogenous interaction between HIF-2α and B-Myb. 786-O cell lysates were immunoprecipitated (IP) with an anti-HIF-2α antibody and immunoblotted with anti-B-Myb or anti-HIF-2α antibodies. Representative data from three independent experiments are shown. (B) Cytoplasmic and nuclear fractions were prepared from 786-O cell lysates and utilized for co-immunoprecipitation assays as in (A). Endogenous HIF-2α and B-Myb are enriched in the nucleus but also localize to the cytoplasm. An interaction between HIF-2α and B-Myb was detected in the nuclear fraction but not the cytoplasmic fraction. Hsp90 and Lamin B1 were used as cytoplasmic and nuclear protein markers, respectively. Representative data from two independent experiments are shown.

As HIF-2α was considered to stabilize B-Myb, we examined the interaction between B-Myb and HIF-2α (Fig 3A). Endogenous HIF-2α was immunoprecipitated using an anti-HIF-2α antibody and B-Myb was immunoblotted with an anti-B-Myb antibody. As a result, B-Myb was shown to be co-immunoprecipitated with HIF-2α (Fig 3A). The reciprocal IP did not give a clear result, which suggests that the epitope recognized by the anti-B-Myb antibody might be masked by HIF-2α. Since both HIF-2α and B-Myb are distributed nucleocytoplasmically [11], we investigated whether their interaction was subcellular localization-dependent, using nuclear and cytoplasmic fractions of 786-O cells in immunoprecipitation assays. An interaction between endogenous HIF-2α and B-Myb was detected in nuclear fractions but not in cytoplasmic fractions (Fig 3B); however, because B-Myb expression levels are significantly lower in the cytoplasm than in the nucleus, we cannot exclude the possibility that these two proteins also interact in the cytoplasm (Fig 3B).

**Fig 4:**
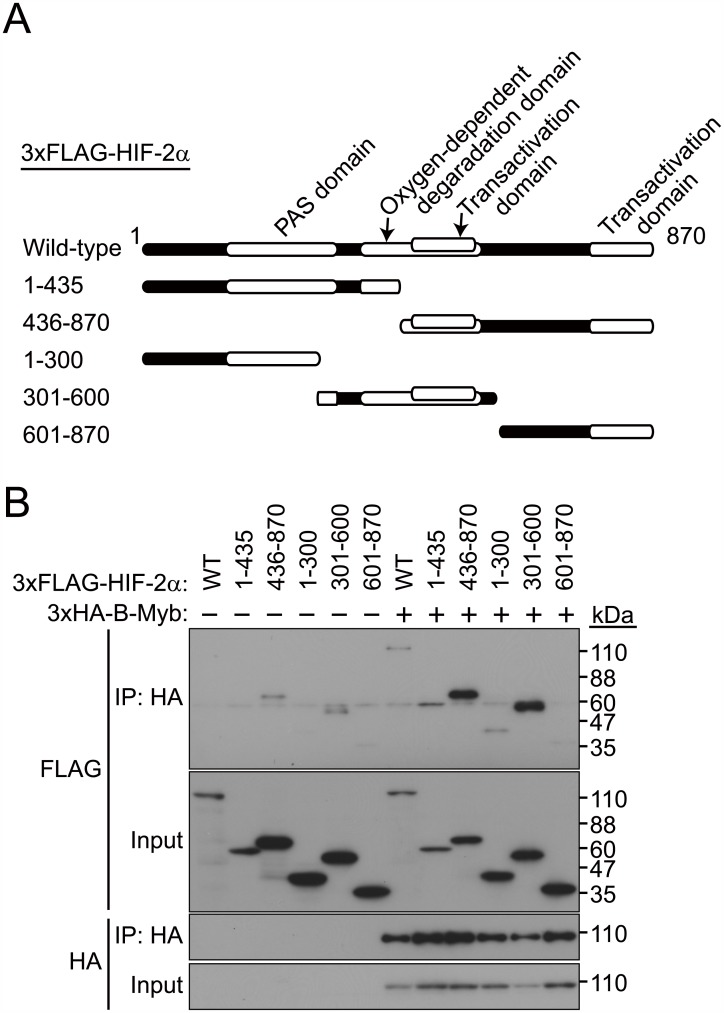
A central region in HIF-2α interacts with B-Myb. (A) Schematic representation of the HIF-2α deletion mutants used in this study. (B) Co-immunoprecipitation between full-length B-Myb and deletion mutants of HIF-2α. Wild-type or deletion mutants of 3×FLAG-HIF-2α were coexpressed with full-length 3×HA-B-Myb in HEK293T cells, and cell lysates were subjected to immunoprecipitation (IP) with an anti-HA antibody and immunoblotted with anti-FLAG or anti-HA antibodies. Deletion mutants of HIF-2α containing a central region between residues 436 to 600, which fully spans the transactivation domain and partially spans the oxygen-dependent degradation domain, interact with full length B-Myb. Representative data from three independent experiments are shown.

**Fig 5:**
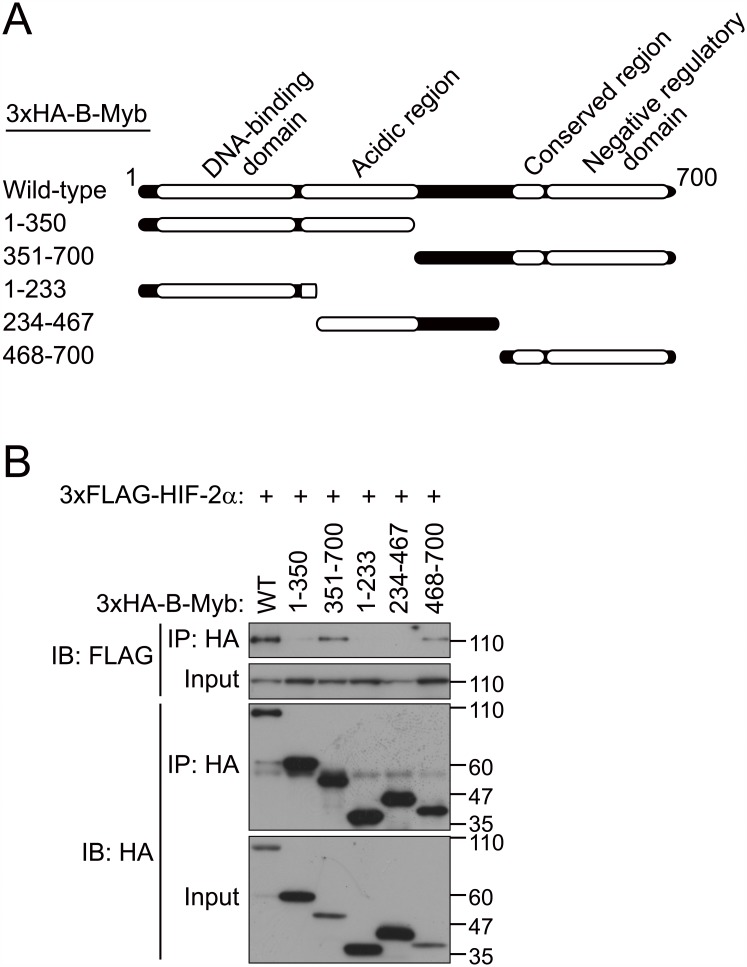
The C-terminal region of B-Myb interacts with HIF-2α. (A) Schematic representation of the B-Myb deletion mutants used in this study. (B) Co-immunoprecipitation between full-length HIF-2α and deletion mutants of B-Myb. Wild-type or deletion mutants of 3×HA-B-Myb were coexpressed with full length 3×FLAG-HIF-2α in HEK293T cells, and cell lysates were subjected to immunoprecipitation (IP) with an anti-HA antibody and immunoblotted with anti-FLAG or anti-HA antibodies. Deletion mutants of B-Myb containing the C-terminal region (starting at residue 468), which comprises the conserved region and the negative regulatory domain, interact with full-length HIF-2α. Representative data from three independent experiments are shown.

To determine the regions of HIF-2α and B-Myb required for their interaction, we constructed a series of deletion mutants of HIF-2α and B-Myb and performed co-immunoprecipitation assays (Figs 4 and 5). Because 3×FLAG-HIF-2α(1–435) expression was weaker than that of 3×FLAG-HIF-2α(436–870), the affinity between these two mutants and B-Myb (Fig 4A and 4B) could not be compared. Co-immunoprecipitation of full-length B-Myb with a series of HIF-2α deletion mutants indicated that a region between residues 436 and 600 in HIF-2α, which fully spans the transactivation domain and partially spans the oxygen-dependent degradation domain, mediates its interaction with B-Myb (Fig 4A and 4B). Co-immunoprecipitation of full-length HIF-2α with a series of B-Myb deletion mutants indicated that the C-terminal region of B-Myb (starting after residue 468), which comprises the conserved region and the negative regulatory domain, mediates its interaction with HIF-2α (Fig 5A and 5B). These results demonstrate that HIF-2α interacts with B-Myb through distinct binding regions on each protein.

### Effect of HIF-2α on B-Myb-dependent gene expression

**Fig 6:**
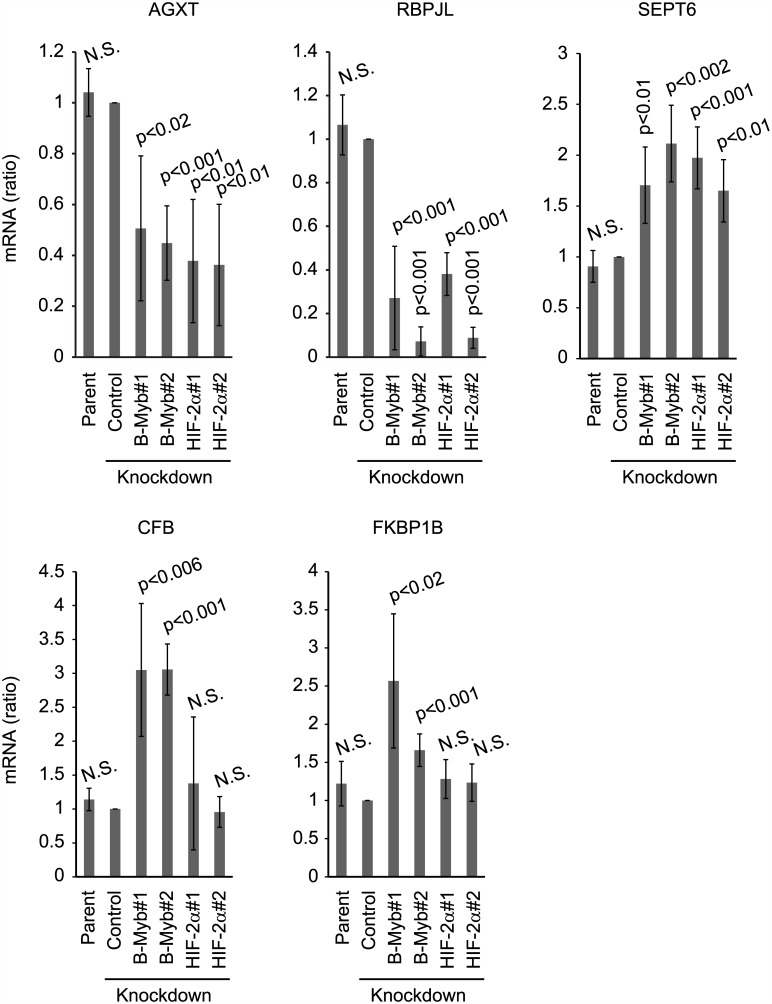
HIF-2α regulates B-Myb-dependent gene expression. Relative mRNA levels of AGXT, RBPJL, CFB, FKBP1B, and SEPT6 were quantified after non-specific control knockdown or knockdown of B-Myb or HIF-2α in 786-O cells. Total RNA was purified from parental 786-O cells, non-specific control, or B-Myb- or HIF-2α-knockdown 786-O cells and analyzed by quantitative RT-PCR. Data represent the mean ± SD of four independent experiments.

To determine whether the interaction between B-Myb and HIF-2α affects B-Myb-dependent gene expression, we investigated the effect of B-Myb and HIF-2α knockdown on the gene expression of alanine-glyoxylate aminotransferase (AGXT) and recombination signal binding protein for immunoglobulin kappa J region-like (RBPJL), which are positively regulated by B-Myb, as well as complement factor B (CFB), FK506 binding protein 1B (FKBP1B), and septin 6 (SEPT6), which are negatively regulated by B-Myb [11]. Knockdown of B-Myb in 786-O cells decreased the mRNA levels of AGXT and RBPJL and increased that of CFB, FKBP1B, and SEPT6, consistent with previous findings (Fig 6). If downregulation of B-Myb by knockdown of HIF-2α has the same effect as B-Myb-specific knockdown of B-Myb-regulated gene expression, and HIF-2α does not affect the expression of these genes, the mRNA levels of AGXT and RBPJL should be decreased by HIF-2α knockdown, and that of CFB, FKBP1B, and SEPT6 should be increased by HIF-2α knockdown. As expected, the mRNA levels of AGXT and RBPJL were decreased by HIF-2α knockdown, and that of SEPT6 was increased by HIF-2α knockdown. In contrast, knockdown of HIF-2α had no effect on the mRNA levels of CFB and FKBP1B (Fig 6), indicating that although HIF-2α regulates B-MyB protein stability, the functional impact on B-Myb target gene expression is limited. Based on the HIF-2α ChIP-Seq data for 786-O cells [15], HIF-2α does not appear to bind within the promoter region of these genes. Therefore, HIF-2α probably regulates gene expression of *CFB* and *FKBP1B* indirectly. Altogether, these results suggest that HIF-2α binds to and stabilizes B-Myb. Furthermore, some B-Myb-dependent gene expression was similarly regulated by knockdown of B-Myb or HIF-2α.

## Discussion

Given that HIF-2α and B-Myb protein levels were increased in the presence of pVHL under hypoxic conditions (Fig 2A), we conclude that HIF-2α prevents the degradation of B-Myb by pVHL. Since B-Myb does not contain an oxygen-dependent degradation domain, HIF-2α may prevent the interaction between pVHL and B-Myb, or ubiquitination of B-Myb. Further investigation is required, however, to identify the exact molecular mechanism of B-Myb stabilization in the presence of HIF-2α, and the contribution of ubiquitin ligase to B-Myb degradation in the absence of pVHL.

B-Myb positively regulates the expression of AGXT and RBPJL and negatively regulates the expression of CFB, FKBP1B, and SEPT6 (Fig 6); therefore, a decrease in B-Myb levels due to downregulation of HIF-2α is expected to regulate the expression of these genes. As expected, knockdown of HIF-2α had a similar effect on the mRNA expression levels of AGXT, RBPJL, and SEPT6 as on B-Myb. In contrast, we found that depletion HIF-2α had no effect on CFB and FKBP1B mRNA expression levels (Fig 6). As gene expression is seldom regulated by a single transcription factor, these findings suggest alternative pathways regulating the expression of these genes in HIF-2α knockdown cells. Importantly, how these genes are involved in VHL disease pathogenesis is unknown. It is known that AGXT converts alanine and glyoxylate to pyruvate and glycine for glyoxylate detoxification. *AGXT* mutation leads to primary hyperoxaluria type I (PH1), which is a rare disorder with deposition of calcium oxalate crystals primarily in the urinary tract [16].

RBPJL is required for normal pancreatic development [17]. RBPJ forms trimeric pancreas transcription factor 1 complex (PTF1-J) during the early stage of pancreatic development. Along with the development of pancreatic acinar cells, RBPJL gradually replaces RBPJ and forms PTF1 containing RBPJL (PTF1-L). Acinar differentiation was incomplete in RBPJL-deficient mice and the expression of genes that encode digestive enzymes or proteins regulating exocytosis and mitochondrial metabolism was decreased. Thus, replacement of RBPJ by RBPJL in the PTF1 complex drives acinar differentiation.

Acute inflammation prevents tumor development, whereas chronic inflammation facilitates tumorigenesis [18,19]. In particular, CFB accelerates tumor invasion and migration [18]. Complement activation leads to chronic inflammation, the establishment of an immunosuppressive microenvironment, angiogenesis, and cancer-related signaling [19].

FKBP1A and FKBP1B are cis-trans prolyl isomerases and regulate protein folding [20]. Their homology is approximately 85% and they show almost identical structure [21]. Given that FKBP1A is a component of the EGFR/FKBP1A/HIF-2α pathway, which plays a role in childhood high-grade astrocytomas [22], increased FKBP1B levels may also cause astrocytomas. There are 13 septins (GTP-binding protein, SEPT1-12 and 14) in humans that exist in 6–8 subunit complexes [23]. SEPT6 forms a complex with SEPT2 and SEPT7 (SEPT7- SEPT6- SEPT2- SEPT2- SEPT6- SEPT7), resulting in the formation of the septin filament [24]. Septins also form ring-like structures (septin rings) that regulate cell division, ciliogenesis, and neuronal synapse formation [23]. Altogether, although there are some indications as described above, the underlying molecular mechanism of how these genes contribute to VHL disease remains to be identified.

Our results suggest a putative model wherein the expression of B-Myb is regulated post-transcriptionally by HIF-2α. B-Myb is targeted by pVHL for proteasomal degradation. However, the accumulation of HIF-2α and B-Myb in the absence of pVHL after proteasome inhibition indicates that both proteins are still targeted for proteasomal degradation, although to a lesser extent (Fig 1C). Thus, both proteins are potentially targeted by other ubiquitin ligases in addition to pVHL. Identification of this alternative ubiquitin ligase pathway would offer another means of downregulating HIF-α and preventing VHL pathogenesis. Stabilization of B-Myb by HIF-2α likely functions as a tumor suppressor mechanism in normal physiology. In fact, B-Myb expression is significantly downregulated in human clear cell renal cell carcinoma, as observed in two independent analyses [25,26]. However, the mechanisms by which B-Myb suppresses tumor initiation and growth have not been fully elucidated. Nevertheless, these studies suggest that the genetic or epigenetic downregulation of B-Myb in the absence of functional pVHL facilitates tumor initiation and growth, and that a drug that stabilizes B-Myb may be used to potentially treat renal cell carcinomas.

## Conclusions

As a conclusion, the oncogenic transcription factor HIF-2α was suggested to stabilize VHL disease suppressor B-Myb, which is also a transcription factor, by physical interaction. Some B-Myb-dependent gene expression was similarly affected by B-Myb or HIF-2α knockdown, suggesting that stabilization of B-Myb by HIF-2α may play a role in specific gene expressions. Thereby, it is important to clarify the exact molecular mechanism to understand VHL disease pathogenesis.

## Materials and methods

### Plasmid construction

Human *B-Myb* (GenBank/EBI accession number: NM_002466), *HIF-2α* (GenBank/EBI accession number: NM_001430), and *pVHL* (GenBank/EBI accession number: NM_000551) cDNA were amplified by polymerase chain reaction (PCR) from a 293T cDNA library and subcloned into pcDNA3-puro or pMX-puro expression vectors [11].

### Gene knockdown with siRNA

Non-specific control knockdown [27] and knockdown of B-Myb were performed as previously described [11]. The target sequences for HIF-2α#1 and HIF-2α#2 were 5′-GAGTGAGATTGAGAAGAATGA-3′ and 5′-GACCTGAAGATTGAAGTGATT-3′, respectively.

### Reagents

CoCl_2_ was purchased from Sigma-Aldrich (St. Louis, MO, USA). Protein A-Sepharose was purchased from GE Healthcare Bioscience (Piscataway, NJ, USA). MG132 was purchased from Calbiochem (San Diego, CA, USA). Lactacystin was purchased from Peptide Institute Inc. (Osaka, Japan).

### Cell culture and transfection

HEK293T and 786-O cell lines were purchased from the American Type Culture Collection and cultured as previously described [11]. HEK293T cells were transfected with the expression plasmid, using polyethyleneimine (PEI) (MW-25K, Polyscience Inc., Warrington, PA, USA), plasmid DNA (μg):PEI (μg) = 1:3. Retroviral infections were performed as described previously [28]. In brief, 786-O cells were incubated in retrovirus-containing culture medium for 2 days and selected using puromycin (5 μg/ml) for 1 week. Additionally, siRNA was transfected into 786-O cells, using Lipofectamine RNAiMAX Reagent (Thermo Fisher Scientific) per the manufacturer’s instructions. At 2 days post transfection, cells were harvested and lysed.

### Antibodies

Antibodies against the following were used in this study: FLAG (1 μg/mL; M2, Sigma-Aldrich), HA (1 μg/mL; 12CA5, Sigma-Aldrich), B-Myb (1 μg/mL; sc-81192, Santa Cruz Biotechnology, Santa Cruz, CA, USA), and HIF-2α (1 μg/mL; sc-13596, Santa Cruz Biotechnology). Rabbit anti-pVHL antibody was also used as described previously [11].

### Fractionation, Immunoprecipitation (IP) and Immunoblot (IB) analyses

Fractionation, IP, and IB analyses were performed as previously described [11].

### Quantitative Reverse Transcriptase (RT)-PCR analysis

Quantitative PCR analysis was performed using the StepOnePlus RT-PCR system (Applied Biosystems, Foster City, CA, USA) and Thunderbird SYBR qPCR mix (Toyobo, Tokyo, Japan). The primer sequences were as follows: human *B-Myb*, 5’-AGTCTCTGGCTCTTGACATTG-3’ and 5’-GGGTGAGGCTGGAAGAGTTTG-3’; human *CFB*, 5’-AAAGCTCTGTTTGTGTCTGAG-3’ and 5’-ATGTCCTTGACTTTGTCATAG-3’; human *FKBP1B*, 5’-GGTTGCAGATTGAAGCATTTC-3’ and 5’-GGCAGTGTAGATTGTGCGAAC-3’; human *SEPT6*, 5’-TGTCAGCAACGGAGTCCAGAT-3’ and 5’-GTGCTGCCAATGACAGCAAAC-3’; human *AGXT*, 5’-CTCCTGGAAACAGTCCACTTG-3’ and 5’-TGGCCAGGCCCTTTATTAAAC-3’; human *RBPJL*, 5’-CCGCCTTATCAAGGTCATCTC-3’ and 5’-GGTTGAAGAGGGAGACCTTTG-3’; human *GAPDH*, 5′-GCAAATTCCATGGCACCGT-3′ and 5′-TCGCCCCACTTGATTTTGG-3′.

### Statistical analysis

The statistical significance of differences between groups was determined by one-way ANOVA. P < 0.05 was considered statistically significant.
